# The Mediating Role of Depression in Association Between Total Sleep Time and Instrumental Activities of Daily Living in China

**DOI:** 10.3389/ijph.2023.1605678

**Published:** 2023-04-04

**Authors:** Yunyi Wu, Sangsang Li, Dan Han, Mei Zhang, Jie Zhao, Hui Liao, Ying Ma, Chaoyang Yan, Jing Wang

**Affiliations:** ^1^ Department of Health Management, School of Medicine and Health Management, Tongji Medical College, Huazhong University of Science and Technology, Wuhan, China; ^2^ The Key Research Institute of Humanities and Social Science of Hubei Province, Huazhong University of Science and Technology, Wuhan, China; ^3^ Institute for Poverty Reduction and Development, Huazhong University of Science and Technology, Wuhan, China

**Keywords:** total sleep time, IADL disability, depression, gender difference, panel data

## Abstract

**Objectives:** This study aims to investigate the mediating role of depression and the moderating effect of gender in the relationship between total sleep time (TST) and instrumental activities of daily living (IADL) in middle-aged and elderly people (aged 45 or above).

**Methods:** The data used in this study is from the China Health and Retirement Longitudinal Study (CHARLS), including a total of 10,460 respondents. Associations between TST, IADL, depression, and gender were analyzed using logistic regression and Karlson, Holm, and Breen (KHB) methods.

**Results:** Short (OR = 1.42, 95% CI = 1.28–1.58 of ≤6 h) and long TST (OR = 1.16, 95% CI = 1.02–1.32 of 8–9 h; OR = 1.35, 95% CI = 1.19–1.54 of >9 h) were both associated with IADL. The mediation effect analyses observed that depression explained 64.80% of the total effect of short TST (≤6 h) and IADL, but was insignificant in long TST (8–9 h and >9 h). Meanwhile, gender has moderating effects on the mediation effect model.

**Conclusion:** The study suggests that health interventions that focused on the dimensions of TST and depression are crucial for preventing functional disability while accounting for gender differences.

## Introduction

Functional disability of the elderly is a global health issue. The WHO estimates about 15% of the world’s population lives with a disability—and this number is growing because of the demographic changes including an aging population and the increase in chronic health conditions worldwide (1). China has one of the fastest-growing aging populations in the world (2, 3), and the WHO predicts that the number of elderly people in China with functional disabilities will increase to 66 million by 2050 (3). Functional disability was defined as self-reports of either needing help or having difficulty with activities of daily living (ADL) or instrumental activities of daily living (IADL) (4). Between the two measurements of functional disability, the IADL contains a higher level of activity for independent living than ADL (5), therefore, impairments of IADL may indicate an early decline in physical functions, and may adversely affect health outcomes in older adults (6).

Many studies have identified influencing factors that are related to IADL, such as socioeconomic status (7) and health status (8). Among these factors, it has been shown that sleep restores body function, renews physical and mental energy, and constitutes an essential part in the lives of people (9). Sleep duration has an inverted U-shaped relationship with health outcomes (10), people with normal sleep duration are less likely to experience health risks than those with shorter or longer sleep duration (11). Previous studies have discussed the relationship between the length of total sleep time (TST) and IADL. Some studies have found that only short (12) or long (13) sleep duration was associated with IADL, however, other studies also found that both sleep durations were related to IADL (14, 15).

Depression is regarded as a common cause of substantial disability in the general population (16). Several studies have found that depression was considered to be one of the leading causes of disability in the world (17, 18), whereas depression as a risk factor predicts a functional decline and disability in elders (19). Studies have found that participants with depressive symptoms at baseline had a higher risk of subsequent functional disability than those without depressive symptoms (20), and depressive symptoms can predict disability in older adults over a 2 years time-span (21). In addition, a longitudinal study reported that people with depression may lead to a decrease in their physical activities (22), thus increasing their difficulty in IADL (23).

Several studies also found that depression was associated with the length of TST (24, 25). A longitudinal study reported that depression scores were highly related to the duration of sleep (26). Short and long sleep durations are significantly associated with an increased risk of depression in adults as indicated by meta-analysis (16). However, other studies have shown that short sleep duration, but not long sleep duration, was associated with depression (27). A prospective study found that shorter sleep duration predicted more severe depression (28). Therefore, it is worth exploring whether the TST has a relationship with IADL and the mediation effect of depression on such a relationship.

Moreover, the gender differences in depression have been well documented. Earlier studies have shown that in old age women are more at risk of depression than men (29). Furthermore, a recent study based on a Chinese elderly population also showed that with the same characteristics, women are more likely to become depressed than men (30). Various studies have investigated the reasons for gender differences in depression, with explanations mainly focused on social (such as marital status, social support, and socioeconomic factors), psychological (such as coping repertoires, and mood amplification), and biological factors (30–32). Investigation is warranted into whether gender could play a moderation role in the mediating effect.

According to literature reviews, it is shown that disability greatly affects one’s quality of life (33), causes a greater risk of mortality (34), and increases the utilization and cost of healthcare (35). The WHO reported that persons with disabilities (PWDs) are often excluded from accessing and receiving everyday health services (36). It is important to know how to prevent IADL by controlling correlative factors. Therefore, this study aims to: 1) find the association between TST and IADL; 2) test whether depression is a mediator of TST and IADL; 3) examine whether gender played a moderating role in the model.

## Methods

### Data and Sample

The China Health and Retirement Longitudinal Study (CHARLS) collected the study samples of Chinese residents ages 45 and above to meet the needs of scientific research on the elderly. The CHARLS questionnaire contains various contents, such as basic personal information, family situation, health status, social security, and so on. The baseline national wave of CHARLS was being fielded in 2011, and surveys were conducted in 150 counties, 450 villages, and committees in 28 provinces, including about 10,257 households, and 17,708 individuals (37).

**FIGURE 1:**
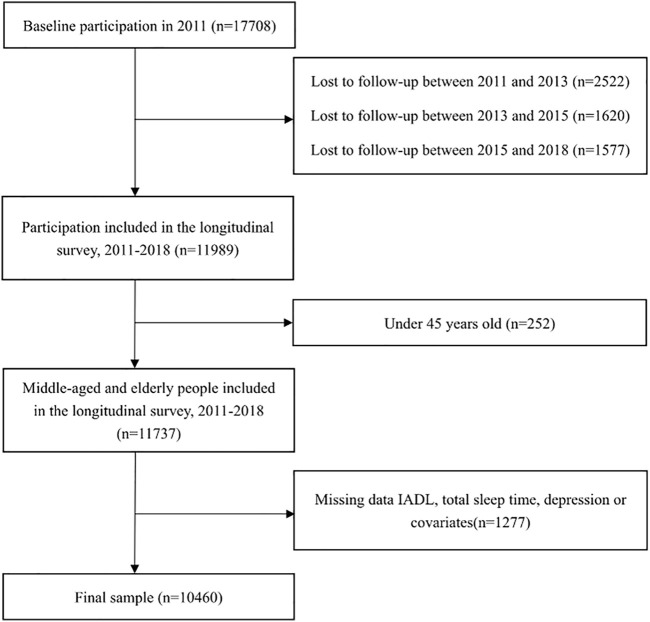
Flowchart of sample selection (China, 2022). Note: IADL, instrumental activities of daily living.

The data used in this study were panel data extracted from four waves of CHARLS conducted in 2011, 2013, 2015, and 2018. At first, the sample included 17,708 participants at baseline. 2,522, 1,620, and 1,577 individuals who were lost to follow-up in wave 2, wave 3, and wave 4, respectively were excluded from the study. Then, 252 individuals whose ages are younger than 45 years in 2011 were also excluded. For the missing values of dependent, independent, and mediating factors, the methods of neighboring and mean value filling were adopted. Samples that still have missing values in the dependent, independent, mediating and any covariates were further removed. The final sample population consisted of 10,460 respondents (Figure 1).

### Variables

#### IADL

The ability to do housework, shop, cook, make phone calls, take medicine, and take care of finances is used to measure the IADL. Each answer was divided into four responses as follows: 1) No, I do not have any difficulty; 2) I have difficulty but can still do it; 3) Yes, I have difficulty and need help; and 4) I cannot do it. In our analysis, participants entered into the IADL disability group if they reported needing help or cannot (i.e., had a response of three or four) conduct one of the 6 IADL items.

#### Total Sleep Time (TST)

Total sleep time (TST) was assessed based on the following questions, “During the past month, how many hours of actual sleep did you get at night (average hours for one night)?” and “During the past month, how long did you take a nap after lunch?” Both nighttime sleep and daytime nap were added together as the TST per day (38). According to other studies in developed countries (39, 40), respondents were categorized into five groups: ≤6 h, >6 to ≤7 h (6–7 h), >7 to ≤8 h (7–8 h), >8 to ≤9 h (8–9 h), and >9 h. Given that an inverted U-shaped relationship between sleep duration and health outcomes has been reported (10), the TST of >7 to ≤8 h was used as the reference group.

#### Depression

Depression was measured by the 10-item Center for Epidemiological Studies Depression Scale (CES-D 10) in the CHARLS questionnaire (37). The CES-D 10 comprised 10 questions about depression, and the answers included four options: 1) rarely; 2) some days (1–2 days per week); 3) occasionally (3–4 days per week); and 4) most of the time (5–7 days per week). Among the 10 questions, eight stated negatively, whereas two stated positively. The answers were recorded as 0 (rarely) to 3 (most of the time) for the negative questions and 3 (rarely) to 0 (most of the time) for the positive questions, respectively. The depression index was obtained from the total scores of the 10 questions. Scores of these 10 items were summed up to create an additive scale score ranging from 0 to 30, with higher scores indicating more depression.

#### Covariates

Covariates included demographic characteristics, socioeconomic status (SES), and health-related variables. A person’s age (year), gender (male, female), race (Han, minority), registered residence (agricultural, non-agricultural), and marital status (married or cohabitated, single) were included in demographic characteristics. Two variables of SES were used: level of education (illiterate, elementary school, middle school, and high school and above), and household income *per capita*. The household income *per capita* was defined as total household income divided by number of people living in this household, and grouped into five categories based on quintile. Person’s chronic conditions, smoking (yes, no), and alcohol use (yes, no) were included in health-related variables. Chronic conditions were measured as the total number of diseases, from a list of 14 diseases, that respondents had been diagnosed with. The number of chronic diseases was coded as 0, 1, 2 and above.

### Statistical Analysis

All statistical analyses were conducted at the individual level and were stratified by gender to explore the differences in the relationships between TST, IADL, and depression among males and females.

First, baseline characteristics of the participants according to gender over 4 years were reported as mean ± standard deviation (SD) for continuous variables and percentages for categorical variables. Descriptive analyses were conducted, and individual characteristics were compared between males and females using the *t*-test (continuous variables) and the Chi-square test (categorical variables).

**FIGURE 2:**
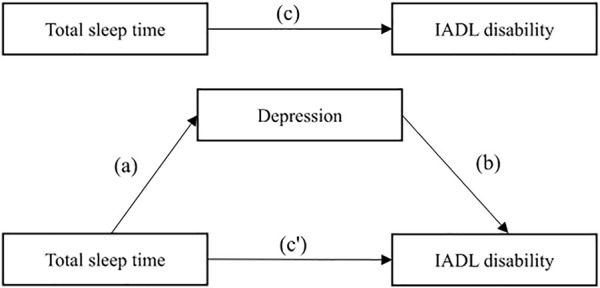
Model of the potential mediating effect of depression on the association between total sleep time and instrumental activities of daily living disability (China, 2022). Note: IADL, instrumental activities of daily living.

Second, the relationship between the three critical variables (TST, depression, and IADL disability) were examined with the logistic regressions command for panel data in Stata, for the full sample, stratified by gender. In this method, X indicates independent variable (TST); Y indicates dependent variable (IADL disability); and M indicates mediator (depression). Binary logistic regression models and multiple linear regression models were used to assess whether variables met the following conditions. First, X is associated with the Y (Figure 2, path c). Second, X is associated with the M (Figure 2, path a). Third, M is associated with the Y after adjusting for X (Figure 2, path b). Finally, X significantly decreases its effect on the Y when M is included in the models as a covariate (Figure 2, path c′); if so, partial mediation is considered to have occurred.

Third, the Karlson, Holm, and Breen (KHB) method is a general decomposition method that is unaffected by the rescaling or attenuation bias that arises in cross-model comparisons in non-linear models, it can decompose the total effect of a variable into a direct and an indirect effect (41). Given the dichotomous nature of the IADL disability measure used in this study and used panel data, the KHB logit model command for panel data in Stata were employed to test the mediation of depression.

Finally, interaction terms to test the moderating effect of gender in the mediation model were added.

All models (adjusted logistic regressions and KHB mediation analyses) were estimated to control the covariate listed in the measurement section. Data processing and analyses were carried out using STATA 16.0, and *p* < 0.05 was considered statistically significant.

## Results

The demographic and health-related characteristics of the study samples were presented in Table 1. A total of 10,460 respondents aged 45 years and above were included in our final population sample, with 4,953 and 5,507 males and females, respectively. At baseline, the average age of the participants was 58.23 (SD = 8.54). Most participants were Han (92.20%), agricultural residents (81.77%), married or cohabitated (90.00%), and received a low level of education (26.28% illiterate and 40.92% elementary school). Women (39.33%) had a higher incidence of multimorbidity (2 and above) than men (34.30%). Men (73.87% and 57.48%) accounted for more of the sample in terms of smoke and alcohol use, than women (7.83% and 12.24%). Descriptive analysis results also showed that there is a gender difference in IADL, TST, depression, and all covariates (*p* < 0.05).

**TABLE 1:** Distribution of baseline (2011) characteristics of the study population by gender (China Health and Retirement Longitudinal Study, China, 2022).

Variables	Total (*n* = 10,460)	Gender		*p*-value
		Male (*n* = 4,953)	Female (*n* = 5,507)	
Age (mean ± SD)	58.23 ± 8.54	58.72 ± 8.49	57.80 ± 8.56	<0.001
Race (%)				0.032
Han	9,644 (92.20)	4,596 (92.79)	5,048 (91.67)	
Minority	816 (7.80)	357 (7.21)	459 (8.33)	
Registered residence (%)				<0.001
Agricultural	8,553 (81.77)	3,958 (79.91)	4,595 (83.44)	
Non-Agricultural	1,907 (18.23)	995 (20.09)	912 (16.56)	
Marital status (%)				<0.001
Married or cohabitated	9,414 (90.00)	4,594 (92.75)	4,820 (87.52)	
Single	1,046 (10.00)	359 (7.25)	687 (12.48)	
Educational level (%)				<0.001
Illiterate	2,749 (26.28)	538 (10.86)	2,211 (40.15)	
Elementary school	4,280 (40.92)	2,267 (45.77)	2,013 (36.55)	
Middle school	2,232 (21.34)	1,364 (27.54)	868 (15.76)	
High school and above	1,199 (11.46)	784 (15.83)	415 (7.54)	
Household income *per capita* (%)				0.020
Quartile 1 (poorest)	2,092 (20.00)	926 (18.70)	1,166 (21.17)	
Quartile 2	2,091 (19.99)	1,003 (20.25)	1,088 (19.76)	
Quartile 3 (average)	2,091 (19.99)	1,022 (20.63)	1,069 (19.41)	
Quartile 4	2,090 (19.98)	983 (19.85)	1,107 (20.10)	
Quartile 5 (richest)	2,096 (20.04)	1,019 (20.57)	1,077 (19.56)	
Chronic conditions (%)				<0.001
0	3,433 (32.82)	1,726 (34.85)	1,707 (31.00)	
1	3,162 (30.23)	1,528 (30.85)	1,634 (29.67)	
2 and above	3,865 (36.95)	1,699 (34.30)	2,166 (39.33)	
Smoke (%)				<0.001
No	6,370 (60.90)	1,294 (26.13)	5,076 (92.17)	
Yes	4,090 (39.10)	3,659 (73.87)	431 (7.83)	
Alcohol use (%)				<0.001
No	6,939 (66.34)	2,106 (42.52)	4,833 (87.76)	
Yes	3,521 (33.66)	2,847 (57.48)	674 (12.24)	
IADL (%)				<0.001
No	9,374 (89.62)	4,551 (91.88)	4,823 (87.58)	
Yes	1,086 (10.38)	402 (8.12)	684 (12.42)	
Total sleep time (%)				<0.001
≤6 h	3,915 (37.43)	1,637 (33.05)	2,278 (41.37)	
6–7 h	1,962 (18.76)	935 (18.88)	1,027 (18.65)	
7–8 h	2,125 (20.32)	1,088 (21.97)	1,037 (18.83)	
8–9 h	1,400 (13.38)	762 (15.38)	638 (11.59)	
>9 h	1,058 (10.11)	531 (10.72)	527 (9.57)	
Depression (mean ± SD)	8.40 ± 6.29	7.30 ± 5.72	9.39 ± 6.60	<0.001

SD, standard deviation; IADL, instrumental activities of daily living.

Table 2 shows the results of the logistic regression of the mediation model stratified by gender. Path a, compared with 7–8 h short TST, was associated with a higher level of depression among all participants ( β = 1.31, *p* < 0.001 of ≤6 h; β = 0.43, *p* < 0.001 of 6–7 h). For path b, after controlling all the covariates and TST, high scores on depression were related to IADL disability (OR = 1.09, 95% CI = 1.08–1.11 among all participants). However, for path c, the results were different in men and women. Among male participants, TST was only related to IADL disability when it was ≤6 h (OR = 1.29, 95% CI = 1.10–1.53). Among female participants, compared with 7–8 h, the TST of the remaining four subgroups was related to IADL disability. After introducing depression into the model (path c′), the OR value of shorter TST on IADL disability decreased (among male participants OR = 1.04, 95% CI = 0.88–1.23 of ≤6 h; among female participants OR = 1.23, 95% CI = 1.08–1.40 of ≤6 h and OR = 1.14, 95% CI = 0.97–1.33 of 6–7 h), thus implying the potential mediating effect of depression on the relationship between TST and IADL disability.

**TABLE 2:** Logistic regression results of path a, b, c, and c′ stratified by gender (China, 2022).

	Path a β ± SE	Path b OR	Path c OR	Path c' OR
Total sleep time		1.09 (1.08, 1.11)***		
≤6 h	1.31 ± 0.08***		1.42 (1.28, 1.58)***	1.14 (1.03, 1.27)*
6–7 h	0.43 ± 0.08***		1.09 (0.97, 1.23)	1.04 (0.92, 1.17)
7–8 h (ref.)				
8–9 h	−0.04 ± 0.09		1.16 (1.02, 1.32)*	1.18 (1.04, 1.35)*
>9 h	−0.02 ± 0.10		1.35 (1.19, 1.54)***	1.38 (1.21, 1.57)***
Male’s total sleep time		1.11 (1.10, 1.12)***		
≤6 h	1.12 ± 0.12***		1.29 (1.10, 1.53)**	1.04 (0.88, 1.23)
6–7 h	0.32 ± 0.10**		0.95 (0.79, 1.15)	0.91 (0.75, 1.10)
7–8 h (ref.)				
8–9 h	−0.02 ± 0.11		0.94 (0.77, 1.16)	0.97 (0.79, 1.19)
>9 h	0.20 ± 0.13		1.11 (0.90, 1.35)	1.13 (0.92, 1.38)
Female’s total sleep time		1.08 (1.07, 1.09)***		
≤6 h	1.44 ± 0.11***		1.53 (1.34, 1.74)***	1.23 (1.08, 1.40)**
6–7 h	0.54 ± 0.12***		1.21 (1.03, 1.41)*	1.14 (0.97, 1.33)
7–8 h (ref.)				
8–9 h	−0.07 ± 0.13		1.36 (1.15, 1.61)***	1.37 (1.16, 1.63)***
>9 h	−0.23 ± 0.15		1.57 (1.33, 1.87)***	1.60 (1.34, 1.89)***

Adjusted for age, race, registered residence, educational level, marital status, chronic conditions, smoke, alcohol use and household income *per capita*; SE, standard error; OR, odds ratio; *p < 0.05, **p < 0.01, ***p < 0.001.

Table 3 shows the total, direct and indirect (through depression) effect of the TST on the IADL disability and the mediated percentage that depression accounts for. The mediating effect in TST was ≤6 h among all subgroups. When TST was ≤6 h, depression explained 85.70% of the relationship between TST and IADL disability in men (OR = 1.29, 95% CI = 1.22–1.36), while in women, depression explained 53.96% of the relationship (OR = 1.27, 95% CI = 1.21–1.34). When TST was 6–7 h, the mediating effect of depression explained 29.51% of the relationship in women (OR = 1.06, 95% CI = 1.01–1.10), but was not statistically significant in men. Furthermore, the mediating effect of depression on TST and IADL disability was not statistically significant in the long TST (8–9 h and >9 h).

**TABLE 3:** Mediation analyses of the association between total sleep time and instrumental activities of daily living disability by depression stratified by gender (the Karlson, Holm, and Breen Method, China, 2022).

Total sleep time	Total OR (95% CI)	*p*-value	Gender			
			Male OR (95% CI)	*p*-value	Female OR (95% CI)	*p*-value
≤6 h						
Total effect	1.46 (1.32, 1.62)	<0.001	1.34 (1.14, 1.58)	<0.001	1.56 (1.37, 1.78)	<0.001
Direct effect	1.14 (1.03, 1.27)	0.011	1.04 (0.88, 1.23)	0.622	1.23 (1.08, 1.40)	0.002
Indirect effect	1.28 (1.23, 1.33)	<0.001	1.29 (1.22, 1.36)	<0.001	1.27 (1.21, 1.34)	<0.001
Mediated (%)	64.80		85.70		53.96	
6–7 h						
Total effect	1.09 (0.97, 1.23)	0.153	0.96 (0.79, 1.16)	0.648	1.20 (1.03, 1.40)	0.023
Direct effect	1.04 (0.92, 1.17)	0.549	0.91 (0.75, 1.10)	0.323	1.14 (0.97, 1.33)	0.108
Indirect effect	1.05 (1.02, 1.09)	0.002	1.05 (1.00, 1.11)	0.038	1.06 (1.01, 1.10)	0.018
Mediated (%)	NA		NA		29.51	
8–9 h						
Total effect	1.16 (1.02, 1.32)	0.026	0.94 (0.77, 1.16)	0.570	1.35 (1.14, 1.60)	<0.001
Direct effect	1.18 (1.04, 1.35)	0.011	0.97 (0.79, 1.19)	0.775	1.37 (1.16, 1.63)	<0.001
Indirect effect	0.98 (0.95, 1.01)	0.217	0.97 (0.93, 1.02)	0.236	0.99 (0.94, 1.03)	0.513
Mediated (%)	NA		NA		NA	
>9 h						
Total effect	1.37 (1.21, 1.56)	<0.001	1.12 (0.92, 1.37)	0.251	1.59 (1.34, 1.89)	<0.001
Direct effect	1.38 (1.21, 1.57)	<0.001	1.13 (0.92, 1.38)	0.235	1.60 (1.34, 1.89)	<0.001
Indirect effect	1.00 (0.97, 1.03)	0.936	1.00 (0.95, 1.05)	0.874	1.00 (0.96, 1.04)	0.949
Mediated (%)	NA		NA		NA	

Adjusted for age, race, registered residence, educational level, marital status, chronic conditions, smoke, alcohol use and household income *per capita*; The mediated percentage was only calculated in the presence of a significant total and indirect effect (*p* < 0.05); OR, odds ratio; CI, confidence interval.

**FIGURE 3:**
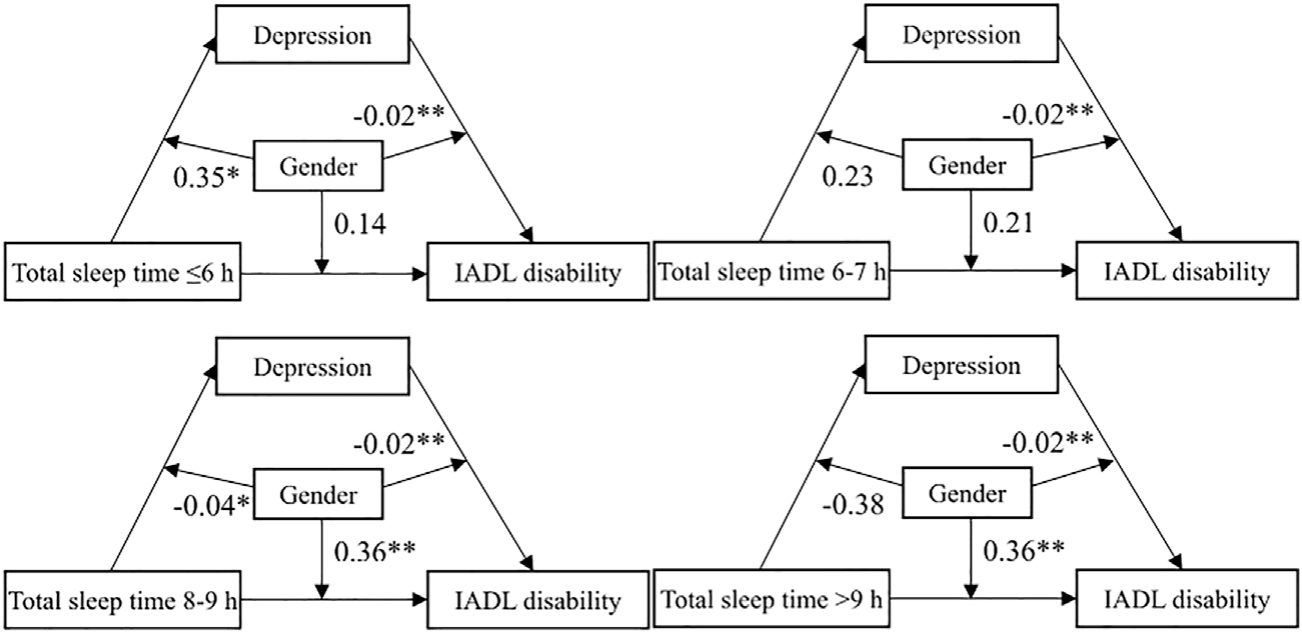
The moderating effect of gender in the mediating effect model (ref. male) (China, 2022). Note: *p < 0.05, **p < 0.01, ***p < 0.001; IADL, instrumental activities of daily living.

Figure 3 shows the moderating effect of gender in the mediating effect model. The moderating effect of depression by gender on IADL disability was significant ( β = −0.02, *p* < 0.01), and the associations were stronger for men. Gender moderates the relationship between long TST ( β = 0.36, *p* < 0.01 of 8–9 h and >9 h) and IADL disability. Compared with men, long TST (8–9 h and >9 h) may have a stronger impact on IADL disability in women. In addition, gender moderates the relationship between TST ( β = 0.35, *p* < 0.05 of ≤6 h; β = −0.04, *p* < 0.05 of 8–9 h) and depression. This may mean that TST has a stronger impact on depression in women at ≤6 h and in men at 8–9 h.

Given the TST in this study was the sum of nap and nighttime sleep, sensitivity analyses were conducted to investigate the relationship between the two types of sleep time and IADL disability and the mediating role of depression. The results of sensitivity analyses showed that the relationship between nighttime sleep and IADL disability and the mediating role of depression were basically consistent with the results of TST. Long and short nighttime sleep were related to IADL disability (OR = 0.39, 95% CI = 0.29–0.50 of ≤5 h; OR = 0.35, 95% CI = 0.21–0.49 of >8 h), and depression explained 64.80% of the total effect of short nighttime sleep (≤5 h) and IADL disability (Supplementary Table S1, S2).

## Discussion

Based on the above results, it was found that TST was associated with IADL disability, and depression had a mediating effect on this association, which was also moderated by gender. It was also found that too long or too short TST was associated with IADL disability, and that short TST was also associated with depression. These results implied that the long TST does not have an indirect effect on IADL disability through depression, whereas short TST indirectly affects IADL disability through depression. These relationships also had significant gender differences. The possible reasons for these results could be related to biological, behavioral, and psychological factors.

### Total Sleep Time and IADL Disability

The relationship between TST and IADL disability may be associated with biological and behavioral factors. In our study, both short (≤6 h) and long (8–9 h and >9 h) TST were associated with IADL disability. Short sleep duration may alter metabolic pathways, thus leading to added insulin resistance and reduced energy expenditure (42). In addition, according to previous studies, possible biological factors also included autonomic nervous system dysregulation (43), metabolic derangement (44), and inflammation (45). Moreover, the behavioral factor was likely to be physical activity. Long sleep duration has been correlated to lack of physical activity, organic disease, and fatigue (46, 47). Therefore, long sleep duration may lead to less physical activity and ultimately develop into IADL disability.

### Mediation of Depression in Short Total Sleep Time and IADL Disability

There is a complex relationship between short TST, IADL disability, and depression. Our research results showed that depression partially explained the relationship between short TST and IADL disability, which are related to psychological factors. Short sleep duration may lead to daytime tiredness (sleepiness and/or psychological fatigue), thus increasing negative events and emotions and eventually predisposing individuals to a risk of depression (48). Several studies have found that short sleep duration was linked to increased depressive symptoms (16). Meanwhile, participants with short sleep duration had a higher risk of depression onset and recurrent depression, as shown in the longitudinal studies of middle-aged and older Chinese adults (49). Elders with depression might experience an amplified burden and increased prevalence of multimorbidity (50), while multimorbidity may increase the risks of disability (51). Furthermore, depression increased cognitive load (52), which may affect physical function. Therefore, short TST may affect depression through psychological factors and further affect physical function leading to IADL disability.

In addition, the relationship between depression mediated short TST and IADL disability may also be related to biological factors. Some scholars have suggested that the association between short sleep duration and depression was related to increased pro-inflammatory cytokines, which are found in both short sleep duration and depression (53). Biological changes associated with depression may increase the risks of developing disability, such as elevated cortisol levels and insulin resistance (54). A meta-analysis also found that the levels of inflammatory cytokines are higher in participants with depressive disorder (55), and elevated markers of inflammation are associated with worse physical function (56). Therefore, short TST may affect IADL disability through depression.

### Gender Differences in the Relationship Between Total Sleep Time and IADL Disability

Gender varies the effects of TST on IADL disability, and this may be related to gender differences in behavior. Our research results show that short TST was associated with IADL disability in both men and women, whereas long TST was associated with IADL disability for women only. In a Finnish study, women had a longer preferred sleep duration than men (57). An American study showed that women slept more than men (9). This may mean that women generally need more sleep and actually sleep longer than men. However, various evidence was gathered that women get less high-quality and uninterrupted sleep (58, 59). The compensation for sleep loss was not sufficient, thus could lead to an accumulation of sleep loss especially in women (57). This may imply that long TST still was associated with IADL disability despite women having longer sleep duration.

### Gender Differences in the Relationship Between Depression and IADL Disability

Gender has different effects of depression on IADL disability, this may also be related to behavioral factors. The results showed that there was a gender moderating effect between depression and IADL disability. Earlier studies have shown that older women are at a greater risk of depression than their male counterparts (29, 30), which increases their difficulty in IADL (23). However, our research unexpectedly found that the coefficients for gender (ref. men) in depression and IADL disability were negative. This may mean that the association between depression and IADL disability is stronger in men. Some researchers considered that women are more likely to admit and complain about their dysphoric feelings than men (60, 61), and women are more likely to seek help (31). This may indicate that depression in women was more likely to be overestimated because of these behavioral factors. Therefore, in reality, men may have a stronger correlation between depression and IADL disability.

### Strengths and Limitations

A major strength of this study was its large sample size from a prospective cohort, consequently giving us a great potential to draw a reasonable conclusion. This study still has several limitations. First, the CHARLS data was used, which provided information on IADL disability and TST, and some covariates were self-reported, which may result in measurement error. However, this self-reported information was also in other large population-based studies and shows fairly good specificity and positive predictive values (14), which indirectly supported the validity of the information. Second, to judge the degree of depression this study used CES-D rather than a clinical diagnosis, and this may lead to errors in the judgment of depression degree. However, the CES-D has high sensitivity and specificity in monitoring clinically significant depression in older adults (62). Third, this study used balanced panel data, so there was an autocorrelation problem of the same individual in different waves. However, balancing panel data has many advantages, such as controlling the impact of omitted variables and generating more accurate predictions (63). Fourth, this study was to explore the association between TST and IADL, so the results cannot explain the causation. Finally, this work only studied the respondents’ TST and does not consider other factors such as sleep quality, sleep efficiency, etc., and the sleep domains in this study were relatively limited. Given that the data in this study were all from CHARLS, other variables related to sleep were lacking in the database, consequently, we were not able to conduct in-depth research in the field of sleep. Despite these limitations, the results of this study could help us better understand the relationship between TST, IADL disability, depression, and gender.

### Conclusion

Both short and long TST are associated with IADL disability, and depression mediated the relationship between short TST and IADL disability. In addition, gender moderated the relationship between TST, depression, and IADL disability. First, the government should strengthen mental health construction and sleep-related health education, differences in population and gender should also be considered when implementing intervention measures. Second, medical institutions should pay attention to the changes in human biology, which may indicate changes in human health. Finally, individuals should give special attention to health-related behaviors, such as increased physical activity and proper sleep duration and schedules, and should pay attention to their mental state.
