# Supplementary material for: The Mediating Role of Depression in Association Between Total Sleep Time and Instrumental Activities of Daily Living in China
Source: Int J Public Health. 2023 Apr 4;68:1605678. doi: 10.3389/ijph.2023.1605678 (PMC10110912; doi:10.3389/ijph.2023.1605678)
Supplement: Supplementary file 1 [file Table1.DOCX]

**Table S1** Logistic regression results of path a, b, c, c' of nap and nighttime sleep (China, 2023).

|  | Path a $\beta$±SE | Path b OR | Path c OR | Path c' OR |
| --- | --- | --- | --- | --- |
| Nap time |  | 0.09(0.08,0.09)*** |  |  |
| 0 min | -0.00±0.10 |  | 0.13(-0.01,0.26) | 0.10(-0.03,0.23) |
| 0-15 min | -0.12±0.13 |  | 0.39(0.22,0.56)*** | 0.41(0.24,0.58)*** |
| 15-30 min (ref.) |  |  |  |  |
| 30-60 min | -0.06±0.10 |  | 0.13(-0.01,0.27) | 0.15(0.01,0.29)* |
| >60 min | -0.12±0.12 |  | 0.18(0.03,0.33)* | 0.23(0.08,0.37)** |
| Nighttime sleep |  | 0.09(0.08,0.09)*** |  |  |
| ≤5 h | 1.41±0.09*** |  | 0.39(0.29,0.50)*** | 0.16(0.05,0.27)** |
| 5-6 h | 0.34±0.08*** |  | 0.05(-0.07,0.17) | -0.01(-0.13,0.10) |
| 6-7 h (ref.) |  |  |  |  |
| 7-8 h | -0.28±0.08*** |  | 0.05(-0.07,0.17) | 0.08(-0.04,0.20) |
| >8 h | -0.16±0.11 |  | 0.35(0.21,0.49)*** | 0.36(0.22,0.50)*** |

Note:

Adjusted for age, race, registered residence, educational level, marital status, chronic conditions, smoke, alcohol use and household income per capita;

SE, standard error; OR, odds ratio;

*p<0.05, **p<0.01, ***p<0.001.
