# Supplementary material for: The Mediating Role of Depression in Association Between Total Sleep Time and Instrumental Activities of Daily Living in China
Source: Int J Public Health. 2023 Apr 4;68:1605678. doi: 10.3389/ijph.2023.1605678 (PMC10110912; doi:10.3389/ijph.2023.1605678)
Supplement: Supplementary file 2 [file Table2.DOCX]

**Table S2** Mediating effect analysis of depression between sleep time (divided into nap and nighttime sleep) and instrumental activities of daily disability (China, 2023)

|  | Total effect  OR (95% CI) | P value | Direct effect  OR (95% CI) | P value | Indirect effect  OR (95% CI) | P value | Mediated  (%) |
| --- | --- | --- | --- | --- | --- | --- | --- |
| Nap time |  |  |  |  |  |  |  |
| 0 min | 1.14(1.00,1.30) | 0.058 | 1.10(0.97,1.26) | 0.148 | 1.03(0.99,1.07) | 0.146 | NA |
| 0-15 min | 1.49(1.25,1.77) | <0.001 | 1.51(1.27,1.77) | <0.001 | 0.98(0.94,1.03) | 0.451 | NA |
| 15-30 min (ref.) |  |  |  |  |  |  |  |
| 30-60 min | 1.13(0.98,1.29) | 0.096 | 1.16(1.01,1.33) | 0.037 | 0.97(0.93,1.01) | 0.150 | NA |
| >60 min | 1.20(1.03,1.39) | 0.016 | 1.25(1.08,1.45) | 0.003 | 0.96(0.92,1.00) | 0.032 | -24.90 |
| Nighttime sleep |  |  |  |  |  |  |  |
| ≤5 h | 0.44(0.33,0.55) | <0.001 | 0.16(0.05,0.27) | 0.005 | 0.28(0.24,0.32) | <0.001 | 64.18 |
| 5-6 h | 0.07(-0.05,0.19) | 0.266 | -0.01(-0.13,0.10) | 0.811 | 0.08(0.05,0.11) | <0.001 | NA |
| 6-7 h (ref.) |  |  |  |  |  |  |  |
| 7-8 h | 0.06(-0.06,0.18) | 0.299 | 0.08(-0.04,0.20) | 0.198 | -0.02(-0.05,0.02) | 0.353 | NA |
| >8 h | 0.37(0.23,0.51) | <0.001 | 0.36(0.22,0.50) | <0.001 | 0.01(-0.02,0.04) | 0.519 | NA |

Note:

Adjusted for age, race, registered residence, educational level, marital status, chronic conditions, smoke, alcohol use and household income per capita;

The mediated percentage was only calculated in the presence of a significant total and indirect effect (p<0.05);

OR, odds ratio; CI, confidence interval.
